# The Detection of Toxic Amyloid-β Fibril Fragments Through a Surface Plasmon Resonance Immunoassay

**DOI:** 10.3390/ijms252313020

**Published:** 2024-12-04

**Authors:** Marten Beeg, Beatrice Rocutto, Elisabetta Battocchio, Letizia Dacomo, Alessandro Corbelli, Fabio Fiordaliso, Claudia Balducci, Marco Gobbi

**Affiliations:** 1 Laboratory of Pharmacodynamics and Pharmacokinetics, Istituto di Ricerche Farmacologiche Mario Negri IRCCS Via Mario Negri 2, 20156 Milan, Italy; marten.beeg@marionegri.it (M.B.); beatrice.rocutto@marionegri.it (B.R.); elisabetta.battocchio@in.cnr.it (E.B.); 2 Laboratory of Biology of Neurodegenerative Disorders, Istituto di Ricerche Farmacologiche Mario Negri IRCCS Via Mario Negri 2, 20156 Milan, Italy; letizia.dacomo@marionegri.it; 3 Laboratory of Molecular Biology, Istituto di Ricerche Farmacologiche Mario Negri IRCCS Via Mario Negri 2, 20156 Milan, Italy; alessandro.corbelli@marionegri.it (A.C.); fabio.fiordaliso@marionegri.it (F.F.)

**Keywords:** Alzheimer’s disease (AD), Amyloid-β (Aβ), Aβ fibril fragmentation, immunoassay, surface plasmon resonance (SPR), neurotoxicity

## Abstract

Amyloid-β1–42 (Aβ42) forms highly stable and insoluble fibrillar structures, representing the principal components of the amyloid plaques present in the brain of Alzheimer’s disease (AD) patients. The involvement of Aβ42 in AD-associated neurodegeneration has also been demonstrated, in particular for smaller and soluble aggregates (oligomers). Based on these findings and on genetic evidence, Aβ42 aggregates are considered key players in the pathogenesis of AD and targets for novel therapies. Different approaches are currently used to detect the various aggregation states of Aβ peptide, including spectrophotometric methods, imaging techniques, and immunoassays, but all of these have specific limitations. To overcome them, we have recently exploited the peculiar properties of surface plasmon resonance (SPR) to develop an immunoassay capable of selectively detecting monomers and oligomers, discriminating them also from bigger fibrils in a mixture of different aggregated species, without any manipulation of the solution. In the present study, we extended these previous studies, showing that the SPR-based immunoassay makes it possible to unveil the fibril fragmentation induced mechanically, a result difficult to be conveniently and reliably assessed with other approaches. Moreover, we show that SPR-recognized fibril fragments are more toxic than the larger fibrillar structures, suggesting the relevance of the proposed SPR-based immunoassay.

## 1. Introduction

One of the main hallmarks of Alzheimer’s disease (AD) is the presence of senile plaques in the brain, composed of insoluble deposits of abnormally folded β-amyloid (Aβ), an aggregation-prone fragment of 39–43 amino acids, most commonly Aβ1–40 (Aβ40) and Aβ42, formed through the proteolytic cleavage of the Amyloid Precursor Protein (APP).

Aβ40/42 monomers exhibit a high tendency to aggregate, forming small soluble oligomers at first, which subsequently progress to larger and insoluble amyloid fibrils, which can further assemble into amyloid plaques. Fibrils present a cross-β structure, in which β-strands are oriented perpendicularly to the fibril axis and are assembled into β-sheets that run the length of the fibrils [1].

Oligomeric species are not just precursors of insoluble fibrillar deposits, but they are the most toxic species in AD, based on a large bulk of evidence. For instance, Aβ oligomers contribute to the loss of dendritic spines, which play a crucial role in short-term memory formation and maintenance, as well as a gradual decrease in hippocampal synapses [2]. Oligomeric species also inhibit long-term potentiation (LTP), indicating an impact on synaptic plasticity [3], impair memory through mutual collaboration with glial cells [4,5], cause cellular dysfunction [6], and trigger neuronal death [7]. On these bases, as well as on genetic evidence, Aβ aggregates—and in particular, Aβ oligomers—are considered key players in the pathogenesis of AD.

However, the term “oligomers” is broad and encompasses various species of small, soluble Aβ aggregates. Oligomers include unordered species formed in the initial aggregation steps, which later undergo conformational changes to form the typical β-sheet fibrillar structures [8,9]. However, they could also include short protofibrillar aggregates, which appear early in the aggregation process or form upon fibril fragmentation induced purposely by drugs or other treatments (e.g., irradiation). The toxicity of oligomeric species, in particular, the unordered ones, is linked to their small size, the exposure of highly reactive hydrophobic groups on their surface, and their high diffusion coefficient, which enables them to move more rapidly and form abnormal interactions [10,11]. While fibril fragmentation can reduce the bulk toxicity of large aggregates, the resulting smaller fragments may expose reactive fibrillar ends that facilitate elongation, accelerating further aggregation and potentially increasing harmful interaction with cells [12].

In order to better characterize the mechanisms underlying AD progression and to develop novel therapies, it is essential to have suitable methods to identify and classify the various species of Aβ. These methods are required, for instance, to analyze the kinetics of oligomers and fibril formation under different conditions or to evaluate the effects of potential drugs as aggregation inhibitors or destabilizers of specific aggregation states. Notably, detecting and analyzing oligomers is particularly challenging, as their low stability and heterogeneous nature make this task very difficult.

The main available approaches include:

- Microscopy techniques, such as transmission electron microscopy (TEM) and cryogenic electron microscopy (Cryo-EM), allow for the direct visualization of different aggregated forms. These methods provide qualitative information by enabling the morphological characterization of fibrils and oligomers. However, they are limited by the difficulty and time required for the rigorous quantification of the different species [1,13].
- Techniques based on β-sheet recognition enable the specific and quantitative determination of Aβ fibrillar structures. These include spectrophotometric techniques using dyes, such as thioflavin-T (ThT), which specifically bind to hydrophobic grooves along the length of the fibrils [14], giving a specific spectral response [1,15]. Other techniques such as circular dichroism (CD) and Fourier Transform Infrared Spectroscopy (FTIR), and solid-state Nuclear Magnetic Resonance (ssNMR), which allow for the specific detection of β-sheets and provide detailed structural insights into fibrillar assemblies [16]. While these methods can quantify fibrillar content, they are limited in providing qualitative information about the structure of unordered oligomeric species.
- Biochemical techniques based on immunoassays, such as ELISA, dot blot, and Western blot, provide both qualitative and semi-quantitative data. Some immunoassays exploit anti-Aβ antibodies following centrifugation steps to uniquely measure soluble species [17]; others rely on conformation- or epitope-specific antibodies that could recognize specific aggregate subpopulations [18,19]. However, these techniques require indirect readouts, with long incubation steps, and washing procedures that could interfere with the recognition of transient and unstable species [20].

In order to reliably detect transient oligomeric species in a mixture of aggregated species without sample manipulation, an immunoassay based on surface plasmon resonance (SPR) has been proposed [21]. This method exploits the properties of an anti-Aβ antibody (4G8) for a direct and simultaneous determination of monomers and biologically relevant (i.e., toxic) transient oligomeric species, also distinguishing the latter from large fibrils. SPR is a highly quantitative technique for measuring binding kinetics and determining monomer concentrations. However, in the context of aggregation assays, its role becomes semi-quantitative due to the heterogeneous size and unknown concentrations of aggregates. Nevertheless, the SPR-based approach appeared very suitable for studying the effect of compounds on the different aggregation states. For example, it made it possible to demonstrate that the formation of Aβ42 oligomers is inhibited by epigallocatechin gallate and increased by the Aβ42A2V mutation [21]. However, this relatively simple and semi-quantitative approach had not yet been tested for its ability to identify compounds/conditions capable of destabilizing Aβ42 fibrils, inducing their fragmentation (i.e., with the formation of potentially toxic oligomeric species) or depolymerization (i.e., reverting to monomers).

For this purpose, in the present work, we extensively evaluated the potential of the SPR-based method in detecting fibril fragmentation induced by sonication or freeze-thaw cycles, conducting in parallel classic TEM and ThT analysis and assessing the biological effects of the fragmented fibrils.

## 2. Results

### 2.1. SPR-Based Assay Is Suitable for Detecting Aβ42 Fibril Fragmentation

To evaluate the suitability of the SPR-based assay for detecting Aβ42 fibril fragmentation, 50 µM Aβ42 was incubated in PBS at 37 °C without shaking, and samples were subsequently collected at distinct time intervals: at t = 0 (i.e., freshly prepared Aβ42), to have a solution containing monomers only; at t = 6 h, i.e., to have a solution also containing oligomers/small fibrils [21]; and after a long incubation time, at t = 5 days, chosen to ensure complete equilibrium in fibril formation, resulting in a solution containing mainly compact suprafibrillar macroaggregates. The fibril fragmentation was induced by exposing the 5-day solution to either one freeze-and-thaw cycle or sonication.

**Figure 1:**
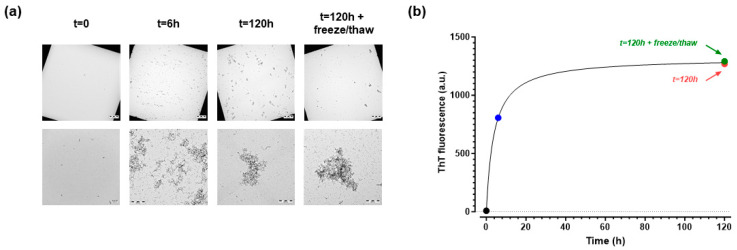
Characterization by TEM (**a**) and ThT (**b**) of the Aβ42 species obtained after different times of incubation at 37 °C, from t = 0 (i.e., freshly prepared solution) to 5 days, the latter subjected or not to a freeze–thaw procedure. Note the different resolutions in the TEM images (scale bar = 20 µm and 500 nm in the upper and lower panels, respectively). ThT data are reported in arbitrary units (a.u.’s). For these studies, we used an Aβ42 prepared in-house starting from the corresponding depsi-peptide (see Section 4). The same solutions were used for both TEM and ThT analyses.

The presence of the different species under these conditions was confirmed by transmission electron microscopy (TEM, Figure 1a), while the fibrillar content was assessed by a thioflavin-T (ThT) assay (Figure 1b). At t = 0, no visible Aβ42 aggregates were detected by TEM, and the ThT assay did not show any signal. After 6 h, TEM analysis revealed small fibrils, sometimes grouped in larger fibrillar aggregates associated with an increase in the ThT fluorescence signal. After 5 days, the fibrillation process was completed, as confirmed by TEM, showing the presence of larger suprafibrillar macroaggregates (Figure 1a), and there was a clear plateau in the ThT signal (Figure 1b). Subjecting this solution to a freeze-and-thaw cycle induced a clear fragmentation of these macroaggregates, as shown in TEM images by the re-appearance of the small fibrils surrounding bigger plaques (Figure 1a). As expected, this fragmentation did not change the β-sheet content, and the ThT signal remained unchanged (Figure 1b).

**Figure 2:**
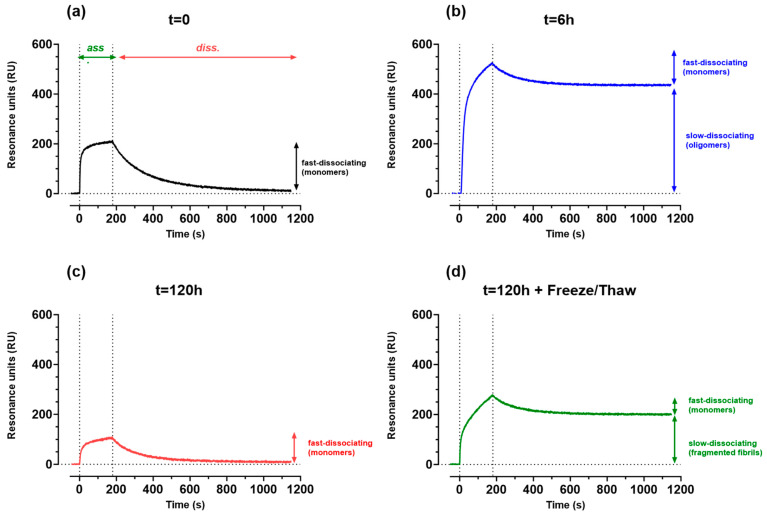
SPR sensorgrams (i.e., time courses of SPR signal in Resonance Units, RUs) obtained by flowing the Aβ42 solutions incubated for different periods of time (0, 6 h and 120 h in panels (**a**–**c**) over 4G8 immobilized on the sensor chip. Aβ42 (5 µM) solutions flowed for 180 s (association phase, highlighted in panel (**a**)) followed by a dissociation phase removing Aβ42 from the running buffer. Panel (**b**) highlights the two binding components, one characterized by a fast dissociation (due to the monomers, as from the data in panel (**a**)) and one characterized by a very slow dissociation (aggregated species). The sensorgram in panel (**d**) was obtained with fibrils obtained after 120 h incubation and exposed to a freeze/thaw fragmentation. For these studies, we used an Aβ42 prepared in-house starting from the corresponding depsi-peptide (see Section 4). The solutions used for these SPR analyses were the same used for the TEM and ThT analyses shown in Figure 1.

The same solutions used for TEM and ThT were flowed in parallel over 4G8 immobilized on the SPR sensor chip, and the corresponding sensorgrams are shown in Figure 2.

The freshly prepared solution (t = 0) showed a clear binding signal (Figure 2a), which was characterized by a very fast association (kon = 3.1 × 10^4^ M^−1^s^−1^), saturation after ≈1 min, and a rapid dissociation (koff = 4.4 × 10^−3^ s^−1^), completing dissociation within 15 min. This binding profile resembles closely that described previously [21] for a solution containing monomers only. The KD calculated from the ratio koff/kon for the Aβ42 monomer binding to immobilized 4G8 was about 140 nM.

The incubation of the Aβ42 solution for 6 h resulted in a marked increase in the SPR binding signal on immobilized 4G8 (Figure 2b). Additionally, the shape of the sensorgram differed significantly from that of the freshly prepared solution (t = 0): in particular, only a small proportion of the bound analyte dissociates during the 15 min dissociation phase (i.e., with the binding properties of monomers), while most of the binding signal showed very slow dissociation (Figure 2b). This aligns with the findings of Stravalaci et al. [21], which also demonstrated that the slowly dissociating species indicate the presence of oligomeric or small fibrillar species. The analysis of these sensorgrams revealed that 6 h of aggregation resulted in a 56% decrease in the monomer signal. The much higher binding signal of the aggregates to 4G8 is likely due to their higher molecular mass and their improved binding properties, mainly for the very slow dissociation rate due to the multivalent binding.

The solution incubated for 5 days produced a much lower SPR signal compared to the solution incubated for 6 h (Figure 2c), and even lower than that of the freshly prepared solution. This signal dissociated completely within 15 min, indicating the presence of residual monomers. Thus, the fibrillar macroaggregates recognized by TEM and ThT (Figure 1) were not recognized by SPR, consistent with previous findings [21]. We hypothesized that this is due to the specific features of SPR technology, in which the flow characteristics in the microfluidic channels may limit the access of large aggregates to immobilized 4G8. The absolute amount of monomers in this solution is similar to the one measured in the solution incubated for 6 h, suggesting that they are in equilibrium with the aggregated species, which evolved from small protofibrils at 6 h to macroaggregates after 5 days.

The exposure of the solution incubated for 5 days to a freeze–thaw cycle resulted in a marked increase in the SPR signals (Figure 2d), and the re-appearance of the slowly dissociating components, likely due to the small aggregates produced by the fragmentation. Interestingly, the absolute amount of monomers decreased, suggesting that the greater number of fibrillar ends produced by fragmentation are still able to elongate by reacting with free monomers.

**Figure 3:**
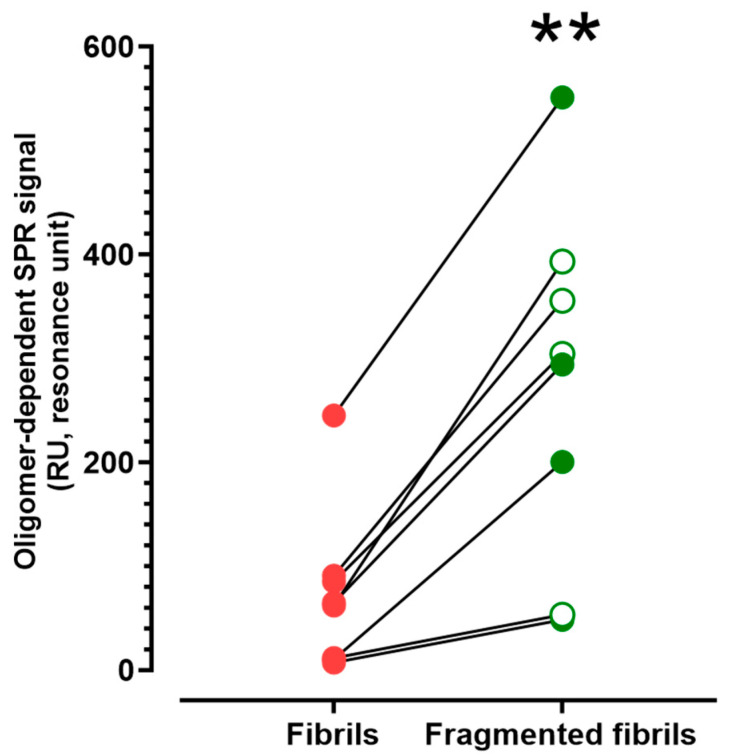
The effect of fibril fragmentation by a freeze–thaw cycle (filled circles) or by sonication (empty circles) on the oligomer-dependent SPR signal (i.e., the one characterized by a slow dissociation, see also Figure 2). The figure shows the results obtained in eight independent experiments, carried out using different Aβ42 (rPeptide, Bachem, or depsi-peptide) or different SPR instruments (Proteon or Bruker, thus explaining the different “basal” values) but always resulting in a fragmentation-induced increase in SPR-detected Aβ42 oligomers. ** *p* = 0.0012 paired *t*-test.

This effect of fibril fragmentation in the SPR assay was consistently observed across eight different independent experimental sessions, using Aβ42 from different sources or exploiting sonication to induce fragmentation (Figure 3).

### 2.2. Toxic Effect of Aβ42 Fibril-Derived Fragments on SHSY-5Y Cells

**Figure 4:**
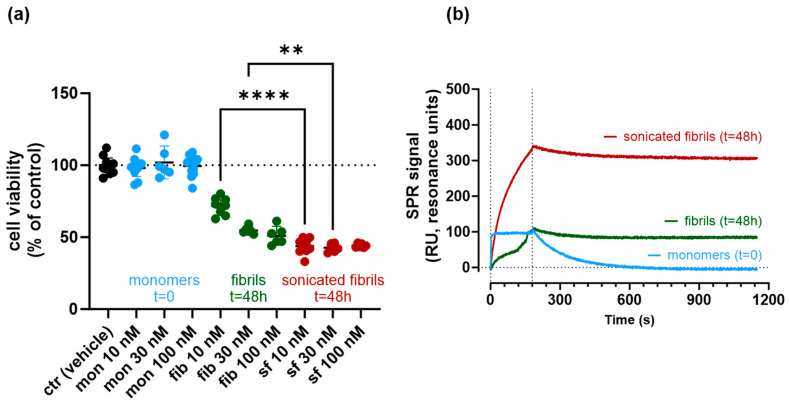
Panel (**a**) Effect of fragmentation (by sonication) on fibril toxicity, as evaluated on SHSY-5Y cells. For these studies, we used an Aβ42 purchased from Bachem, and fibrils were obtained after 48 h incubation at 37 °C (see Section 4). Three independent experimental sessions, each of them in triplicate, were carried out. Aβ42 species (mon = monomers; fib = fibrils; sf = sonicated fibrils) were tested at three different concentrations, from 10 to 100 nM, as indicated. The fact that the fibrillization process was complete is confirmed by the SPR data shown in panel (**b**), which also confirms that fragmentation was effective (compare also with data in Figure 1 and Figure 2). The cell viability measured in the presence of fibrils (±sonication) was significantly different from the viability of control cells treated with vehicle only (ctr, *p* < 0.01,) **** *p* < 0.0001; ** *p* = 0.004 (One-way ANOVA).

The toxicity of Aβ42 fibrils, before and after their fragmentation, was evaluated by exposing SHSY-5Y cells to different concentrations of Aβ42 (from 10 to 100 nM). Figure 4a shows data obtained in three different experimental sessions, highlighting the high reproducibility of our findings. SPR analysis was carried out in parallel in one of them (Figure 4b), confirming the results shown in Figure 2, notably with Aβ42 fibrils prepared from a different source.

The results shown in Figure 4a rule out any toxic effect of the Aβ42 monomers, while fibrils showed concentration-dependent toxicity, already visible at 10 nM, where cell viability was 71.6% of the control (95%CI = 67.3–76.0%). As evident from Figure 4a, fibril toxicity was significantly increased by fragmentation, in particular, at the lowest concentration (cell viability at 10 nM: 43.8% of controls, 95%CI = 39.6–48.0%) but also at 30 nM (42.7% of controls, 95%CI = 39.7–45.71%, versus 54.73% with unfragmented fibrils, 95%CI: 52.1–57.3).

## 3. Discussion

The ability to reliably investigate the disaggregation of preformed Aβ fibrils induced by investigational compounds or conditions is a need for researchers working in this field. The ThT-based assay is one of the most widely used methods for this purpose [22,23,24] due to its simplicity, but this approach has many limitations [25] that are often overlooked. For instance, a decrease in the ThT signal following treatment with a compound might indicate a reduction in β-sheet-containing species (and thus, depolymerization) but could also result from the compound-associated quenching of the ThT fluorescence. The decrease in β-sheet-containing species should be confirmed with other techniques, e.g., circular dichroism, though this is not always feasible since small molecules may interfere with the CD signal. On the other hand, the effects of compounds or conditions that induce fibril fragmentation, resulting in smaller fibrils, are not detectable by ThT assay, as demonstrated in the present study using a freeze–thaw cycle or sonication to induce fibril fragmentation. Imaging techniques, such as TEM or AFM, offer a more objective evaluation of these structural changes and often accompany ThT analysis. However, to ensure objectivity and reliability, these analyses would require a collection of a large number of random images and an accurate image analysis with pre-defined and automated algorithms. This is time- and cost-intensive, particularly for the quantitative analysis of amyloid fibrils, which display high structural variability and very small dimensions, requiring the collection of many images from different areas. Immunoassays are also occasionally exploited, targeting Aβ after the separation of the insoluble fractions, containing the larger fibrils, from the soluble fractions (including smaller species such as protofibrils, oligomers, and monomers). The separation can be achieved by size exclusion, gel electrophoresis, or centrifugation [26,27,28], followed by Western blot or dot blot analysis [19,29,30]. The weakness of these approaches, however, is the need to manipulate the solutions to be tested, which can potentially alter the proportion of the various species. It has been shown that such manipulation may impact sample integrity, potentially affecting the stability and distribution of the species [27,31,32]. Consequently, while all these approaches offer valuable insights, their limitations in quantifying the presence of various species and fibril fragmentation highlight the need for a more reliable quantitative approach.

We previously developed an SPR-based immunoassay, which has the capability to selectively detect Aβ monomers and oligomers, discriminating them also from bigger fibrils, in a short time and, importantly, without any manipulation of the solution.

This method exploits the fact that soluble oligomers bind to chip-immobilized 4G8 in a pseudo-irreversible manner, with very slow dissociation rates likely due to multivalent binding, whereas monomers exhibit significantly faster dissociation rates, and higher-order aggregates are not detectable. The possibility to use 4G8, a general anti-Aβ antibody, is made possible by specific features of SPR, e.g., the fact that it measures the binding events in real-time, in seconds, making it possible to use 4G8 to discriminate between Aβ monomers and oligomers based on their differing dissociation rate constants. Moreover, the lack of any binding of 4G8 to fibrillar macroaggregates makes it possible to discriminate them from smaller aggregates, and this was thought to be due to specific features of the SPR technology that limit large aggregates from reaching the sensor surface. This hypothesis was confirmed in the present study, showing that the mechanical disaggregation of fibrillar macroaggregates results in the “appearance” of an SPR binding signal on immobilized 4G8.

These results establish that the SPR-based 4G8 immunoassay is effective for detecting any destabilization of fibrillar macroaggregates induced by compounds or conditions. In this study, we focused specifically on fibril fragmentation induced by freeze–thaw cycles or sonication, which increases the presence of small, SPR-detectable fibrillar aggregates and, at the same time, decreases monomer concentration, likely for their recruitment at the ends of the fragmented fibrils. Notably, the method would also be capable of detecting fibril depolarization, an event typically associated with an increase in monomer concentration. While the analysis of the rapidly dissociating component of the SPR signal allows for quantitative measurements of monomeric Aβ, the assessment of oligomers is more semi-quantitative in nature. In fact, the slowly dissociating component of the SPR signal depends on both the concentration and degree of oligomerization of the species present: since the distribution of different oligomeric forms is generally heterogeneous and not precisely known, the absolute quantitation of oligomers is not possible. However, the semi-quantitative oligomer readout provided by the SPR sensorgram is still highly valuable for comparative purposes.

While inhibiting oligomer formation may be a promising therapeutic strategy, a number of compounds have been proposed in the literature that bind to amyloid fibrils and whose potential effects on fibril integrity might be detrimental or harmful. In fact, there is evidence that the destabilization of fibrils by natural lipids or their fragmentation by sonication can induce the formation of toxic oligomers [33,34].

Previously, we demonstrated that the transient smaller oligomers, formed during the aggregation process and detectable by immobilized 4G8, are toxic under different experimental models, from cell-free studies to in vivo animal models [21]. Consistent with these findings, this study provides evidence that the fibril fragmentation induced by a freeze–thaw cycle increases toxicity, as evaluated in cells. These results align with Pastor et al. [34], who associated fibril fragmentation by sonication with the creation of harmful oligomers.

## 4. Materials and Methods

### 4.1. Synthesis and Sample Preparation of Aβ42

Aβ42 was selected for this study due to its higher aggregation propensity and its stronger relevance to AD pathology compared to Aβ40, which aggregates more slowly and is less prone to forming the toxic oligomeric and fibrillar structures of interest [35].

Most of the studies were carried out with an Aβ42 peptide produced in-house, starting with the synthesis of depsi-Aβ42 peptide, as described previously [35]. In comparison with the highly aggregating Aβ42, the more soluble depsi form has a much lower propensity for spontaneous aggregation [35,36]. The lyophilized powder obtained from the synthesis and purification was suspended in 0.02% Trifluoracetic acid (TFA). The final pH of the solution was 2.8, ensuring the stability of the depsi bond and preventing premature conversion to the native peptide. The concentration was measured using UV spectroscopy at 214 nm, applying the Lambert–Beer law (molar extinction coefficient = 76,848 M^−^^1^cm^−^^1^) [37]. The stock solution of depsi-Aβ42 in 0.02% TFA was stored at −80 °C in 10 μL aliquots. Once thawed, the aliquots were kept at 4 °C and immediately used.

Native Aβ42 was then obtained from the depsi-peptide by a “switching” procedure involving a change in pH using 10 mM of NaOH [4,35]. We previously demonstrated that a freshly prepared Aβ42 solution obtained in this manner allows the preparation of reproducible, seed-free stock solutions of monomeric Aβ42 [35]. This is routinely checked by circular dichroism, size-exclusion chromatography, and SPR [4,35].

To further validate our results, we used native forms of Aβ42. Lyophilized recombinant Aβ42 (rPepride) and the synthetic Aβ42 (Bachem) were suspended in a 50 mM NaOH solution (1 mg/mL) following the protocol of Taylor et al. [38], which provides a simple and reliable method for the high-yield preparation of seedless Aβ monomers. The stock solutions were stored at −80 °C to preserve the monomeric state, which was shown to remain stable for up to at least 40 days [38]. This validated approach reduces handling variability and ensures reproducibility in experiments.

For experiments, aliquots of the NaOH Aβ42 stock solutions were diluted to a final concentration of 50 or 100 µM in PBS (30 mM phosphate buffer, 137 mM NaCl, pH 7.4) and incubated at 37 °C under quiescent conditions for up to 120 h. This protocol allowed for the controlled aggregation of Aβ42 over time. Samples were collected at specific time points (e.g., 0 h for monomers, 6 h for oligomers/small fibrils, and 2 or 5 days for fibrils) to assess the progression of aggregation [21]. Sonicated Aβ42 fibrils were produced by tip sonication (1 min 10% power 30% cycles) [39].

### 4.2. Surface Plasmon Resonance (SPR)

Surface plasmon resonance (SPR) is a powerful technique used to study real-time interactions between two unlabeled macromolecules. In this setup, one molecule, typically referred to as the “ligand”, is immobilized on a sensor chip, while the other, known as the “analyte”, flows over the immobilized ligand through a microfluidic channel. The interaction is measured in real-time over a very short timeframe [40,41], avoiding lengthy and laborious sample processing steps often required in other techniques, such as ELISA, co-immunoprecipitation, or Isothermal Titration Calorimetry.

For the present work, we used both the ProteOn™ XPR36 Protein Interaction Array system (Bio-Rad Laboratories, Hercules, CA, USA) and the Sierra SPR^®^-32 Pro (Bruker, Billerica, MA, USA). The ProteOn™ XPR36 allows for the preparation of six parallel lanes, each representing a different surface on a single chip, with, for instance, five lanes used for ligand (target) immobilization and one designated as a reference. By rotating the chip, each ligand surface can interact with up to six analytes, enabling the simultaneous analysis of up to 36 unique interactions in a single run [42]. The Sierra SPR^®^-32 Pro is configured with 8 separate channels, each containing four detection spots, one of which serves as a reference spot, enabling analysis with up to 8 samples per run.

The anti-Aβ antibody 4G8 (Signet Laboratories, Emeryville, CA, USA) was immobilized on a CMD50L sensor chip (XanTec bioanalytics, Düsseldorf, Germany) using standard amine coupling chemistry, following standard protocols [21]. A solution of 30 µg/mL 4G8 antibody in 20 mM acetate buffer pH 5.0 was injected for immobilization. The immobilization level was comparable across both instruments, reaching approximately 8000 Resonance Units (RUs, 1 RU corresponds to approximately 1 pg/mm^2^ of molecule bound to the surface [43,44]. For both instruments, a reference surface was prepared by activating and deactivating without antibody immobilization. In the ProteOn™ XPR36, this was done on a parallel empty lane, while in the Sierra SPR^®^-32 Pro, one detection spot per channel was designated as the reference.

To monitor the aggregation of Aβ42 over time, we followed the general protocol described above. Briefly, Aβ42 was prepared at a final concentration of 50 µM in PBS and incubated at 37 °C. Samples were collected at various time points (0, 6, and 120 h) to capture distinct aggregated forms corresponding to monomers, oligomers/small fibrils, and fibrils. These samples were then diluted to 5 µM in PBST (0.005% Tween) and immediately flowed over immobilized 4G8 for 2 min at a flow rate of 30 μL/mL, and dissociation was measured in the next 11 min. The signal from the 4G8-immobilized surface was corrected by subtracting the nonspecific response observed on the empty parallel reference surface.

### 4.3. Transmission Electron Microscopy (TEM)

Transmission electron microscopy (TEM) studies were performed to determine the morphology of the aggregated species formed during the aggregation. Samples were placed on a formvar/carbon-coated copper grid and counterstained with 2% uranyl acetate and observed with an Energy Filter Transmission Electron Microscope Libra 120 (Carl Zeiss AG, Oberkochen, Germany) coupled with an yttrium aluminum garnet scintillator slow-scan charge-coupled device (CCD) camera Sharp eye (TRS, Moorenweis, Germany).

### 4.4. Thioflavin T (ThT) Fluorescence Assay

ThT fluorescence assay is widely used to detect the presence of amyloid fibrils in the solution [45]. For this assay, Aβ42 solutions collected at different time points were diluted to 5 µM in PBS and then mixed with ThT to achieve a final concentration of 20 µM. Fluorescence was measured using an F500 Infinity plate reader (Tecan, Männedorf, Switzerland) with an excitation wavelength of 448 nm and an emission wavelength of 485 nm.

### 4.5. Toxicity

SHSY-5Y cell survival was assessed using the MTT colorimetric assay after exposure to Aβ species—including monomers (t = 0), fibrils, and sonicated fibrils (48 h)—prepared and incubated at a concentration of 100 µM for aggregation. As described in the sample preparation chapter, cells were exposed to these Aβ species at final concentrations of 10, 30, and 100 nM for 24 h. Following exposure, cells were incubated with a culture medium (100 µL/well) containing a 1:10 dilution of Thiazolyl blue tetrazolium bromide (MTT; 4 mg dissolved in 1 mL DPBS). The plates were then placed in the incubator for 3 h. At the end of the incubation, the medium containing MTT was replaced with HCl-Isopropanol (1N HCl diluted 1:25 in 100% isopropanol). The absorbance of the colored solution was measured at 540 nm using a microplate spectrophotometer (F500 Infinity, Tecan, Männedorf, Switzerland).

### 4.6. Statistical Analysis

Statistical calculations were performed using GraphPad Prism (version 9.4; GraphPad Software, Bethesda, MD, USA). Paired *t*-tests were used to analyze differences between experimental conditions (e.g., Figure 3), while one-way ANOVA followed by Tukey’s post hoc test was applied for multiple group comparisons (e.g., Figure 4). The data are presented as mean ± standard deviation (SD). The statistical significance was defined as *p* < 0.05.

## 5. Conclusions

In conclusion, we have shown that SPR-based immunoassay is a valuable tool for studying the dynamics of Aβ aggregation and for rapidly screening and characterizing the effects of potential anti-amyloid agents acting on the various Aβ assembly states. This includes both compounds that prevent aggregation and the formation of toxic oligomers [21] and conditions that induce fibril destabilization (this study, with potential applicability to compound-induced fibril fragmentation). This assay serves a dual purpose: it not only facilitates and supports the development of novel disease-modifying therapies targeting Aβ aggregation but also ensures that any newly developed compounds do not inadvertently generate potentially more toxic oligomeric species. Future work should explore a broader range of small molecules and peptides with potential amyloid-remodeling activity.

## Data Availability

The original contributions presented in this study are included in the article. Further inquiries can be directed to the corresponding author(s).
